# Oral health and oral health-related quality of life in patients with oral dystonia indicates their need for dental special care

**DOI:** 10.4317/medoral.24479

**Published:** 2021-05-23

**Authors:** Gerhard Schmalz, Holger Ziebolz, Tanja Kottmann, Dirk Ziebolz, Rainer Laskawi

**Affiliations:** 1 Department of Cariology, Endodontology and Periodontology, University Leipzig; 2 Private dental practice, Hanau, Germany; 3 Clinical Research Organisation, Hamm, Germany; 4 Department of Otorhinolaryngology, Head and Neck Surgery, University of Göttingen, Göttingen, Germany

## Abstract

**Background:**

This retrospective study aimed in the evaluation of oral health and oral health-related quality of life (OHRQoL) of patients with oral dystonia (OD).

**Material and Methods:**

Seventeen patients with OD (Meige Syndrome: n=11, Oromandibular Dystonia: n=6) were included, of which seven were examined again at three months after botulinum toxin injection. OHRQoL was assessed by the German short form of oral health impact profile (OHIP G14). Within oral examination, dental parameters, remaining teeth and periodontitis severity were assessed. A matched healthy control (HC) was composed for comparison.

**Results:**

The OD patients had significantly more carious teeth (0.94 ± 1.75 vs. 0; *p*<0.01), less remaining teeth (15.65 ± 9.89 vs. 22.22 ± 5.91; *p*=0.01) and higher dental treatment need than the HC (42.9% vs. 0%; *p*<0.01). The OHIP G14 sum score of 9.47± 9.82 vs. 1.58 ± 2.79 (*p*<0.01) as well its dimensions psychosocial impact (4.47 ± 6.45 vs. 0.53 ± 1.16; *p*=0.03) and oral function (4.35 ± 2.98 vs. 0.47 ± 1.34; *p*<0.01) were clinically relevant and statistically significant higher in OD compared to HC group. No significant differences could be detected at three months after botulinum toxin injection.

**Conclusions:**

Patients with OD suffer from more dental diseases and have a worse OHRQoL than HC. Dental special care appears recommendable and should be fostered by everyone, who is involved in the treatment of patients with OD.

** Key words:**Oral health-related quality of life, dental health, oromandibular dystonia, meige syndrome.

## Introduction

Oromandibular Dystonia is characterized as a repetitive and involuntary contraction of the masticatory, facial and/or tongue muscles (1). In case of Meige Syndrom, these symptoms can be complemented by blepharospasm and complex involuntary movement of pharyngeal and cervical muscles (2). The symptoms related to the oral cavity are quite unspecific, reaching from pain, bruxism and tooth fractures to jaw tremor and dislocations/subluxations of the jaw (3). Accordingly, dystonia with oral manifestations are of clinical relevance in dental settings; however, these diseases are rare conditions in dentistry, as a retrospective study over five years showed only 22 patients who entered an orofacial pain clinic to suffer from Oromandibular Dystonia (3).

Available examinations in literature have shown a functional impairment and orofacial pain in these patients (3-5). A case series showed beside of problems with mastication, swallowing and hyposalivation an increase in dental problems (4). However, there is up until now no systematic examination of the dental and periodontal health of these patients. Especially data for patients with Meige Syndrome are still completely missing. Under consideration of the functional and psychosocial burden related to these diseases causing oral dystonia (6), a further oral health associated parameter would be of interest for these patients: the oral health-related quality of life (OHRQoL). This parameter describes the perceived impact of oral diseases and conditions on quality of life and can be seen as a sub-aspect of the whole health-related quality of life of a patient (7). Within four domains, the OHRQoL can allow conclusions on functional, psychosocial, pain-related and aesthetic impairments related to the teeth, mouth or dentures (8). Due to its complexity, the OHRQoL of patients suffering from Oromandibular Dystonia or Meige Syndrome would be of relevance.

Up until now, there is no evidence of potential benefit of oral appliances for treatment of oral dystonia (6). However, the injection of botulinum toxin is repeatedly reported to be a promising therapeutic approach (2,5,9-12). Thereby, beside of functional improvements, also an increase in quality of life has been reported (11). In this context, it is unclear whether functional parameters in combination with OHRQoL can be improved by botulinum toxin, especially over prolonged time period. Furthermore, the oral health situation and a possible necessity of accompanying dental care for these patients is still unclear.

Accordingly, this current study aimed in the evaluation of oral health and OHRQoL of patients with oral dystonia (OD) including patients with Oromandibular Dystonia and Meige Syndrome. Furthermore, it should be examined whether a botulinum toxin injection would lead to improved OHRQoL after three months follow-up. For comparison of oral parameters and OHRQoL, a matched healthy control group was included. It was hypothesized that patients with OD suffer from worse dental health as well as reduced OHRQoL compared to healthy controls.

## Material and Methods

- Study design

This retrospective study was performed to assess oral health and OHRQoL as well as potential influences of a botulinum toxin injection on OHRQoL in patients with OD, including Oromandibular Dystonia and Meige Syndrome.

- Patients

The records of patients suffering from OD, who attended the Department of Otorhinolaryngology, Head and Neck Surgery, University of Göttingen, Göttingen, Germany between 1st April 2012 and 31st December 2014 were included in the current analysis. All participants were part of a special consultation regarding a therapy with botulinum toxin. The patients provided written informed consent for the analysis of their treatment data at the day of first consultation. At this time point, all participants underwent an oral examination in the Department of Preventive Dentistry, Periodontology and Cariology, University Medical Center Goettingen, Germany by the same experienced dentist (HZ), who was previously trained in performing intra- and extra oral examinations of the patients. If possible, participants were examined again at three months after botulinum toxin injection. To participate in the current study, an age of at least 18 years, presence of an Oromandibular Dystonia or a Meige Syndrome as well as complete oral examination data, including OHRQoL assessment were defined as inclusion criteria. Any exclusion criteria did not exist. For comparison, a healthy control group (HC) consisting of 36 patients with comparable age, gender and smoking habits (matching) was composed from the patients of the Department of Preventive Dentistry, Periodontology and Cariology, University Medical Center Goettingen, Germany. In- and exclusion criteria were equal between groups.

- Oral Health Impact Profile

The German short form of oral health impact profile (OHIP G14) was applied as a validated, questionnaire-based tool to assess OHRQoL (13-15). Thereby, 14 questions with regard to functional and psychosocial impacts related to teeth, mouth or dentures were answered on a five-point scale between 0=”never” and 4=”always”. Accordingly, a total score between 0 and 56 points was achievable, with a higher score indicating worse OHRQoL. Beside of statistical significance, clinical relevance was interpreted with regard to the minimal important difference of two points in sum score (16).

- Oral examination

The oral examination comprised of a dental and periodontal investigation. Thereby, the decayed-, missing- and filled teeth index (DMF-T) and the evaluation of periodontal probing depth (PPD) alongside with clinical attachment loss (CAL) were elevated. Within DMF-T, the presence of carious teeth with a cavitation of the tooth surface was assigned to the D-T component (17). Moreover, the number of remaining teeth was recorded. Based on PPD and CAL, measured with a millimetre scaled periodontal probe (PCP 15; Hu-Friedy, Chicago, IL, USA) at six measurement points per tooth, the presence of no/mild, moderate or severe periodontitis was evaluated (18). If at least one carious lesion, deserving invasive therapy measures was apparent, dental treatment need was rated. In case of a PPD ≥ 3.5mm recorded in more than one sextant of the jaw, periodontal treatment need was determined.

Additionally, an examination of the mouth opening (measurement in millimetre) and the evaluation of the axis II Research Diagnostic Criteria for Temporomandibular Disorders (RDC/TMD) (19) was applied at baseline and follow-up for the OD group.

- Statistical analysis

The statistical analysis was performed with SPSS for Windows, version 24.0 (SPSS Inc., US). The metric variables were tested for their normal distribution with Kolmogorov-Smirnov-test. Non-normal distributed samples were analysed by Mann-Whitney-U test. Two dependent variables were analysed by t-test or Wilcoxon test, depending on normal distribution, respectively. The comparison between baseline and follow-up data was performed with chi-square test, modified after Mc-Nemar. The significance level has been set at *p*<0.05.

## Results

- Patients

In the OD group, 17 patients were included, of which about two third suffered from Meige Syndrome (n=11) and six patients were diagnosed with Oromandibular Dystonia (n=6). The HC group consisted of 36 patients with comparable age, gender and smoking habits with the OD group (Table 1). None of the participants had an OD following dental procedures.

- Oral examination

With a value of 22.24 ± 7.45, the DMF-T was significantly higher in the OD compared to HC group (17.64 ± 5.58, *p*=0.01). Furthermore, the OD patients had significantly more carious teeth (0.94 ± 1.75 vs. 0; *p*<0.01) and less remaining teeth (15.65 ± 9.89 vs. 22.22 ± 5.91; *p*=0.01) than the HC group. Accordingly, the dental treatment need was significantly higher in OD group (42.9% vs. 0%; *p*<0.01, Table 2).

**Table 1:**
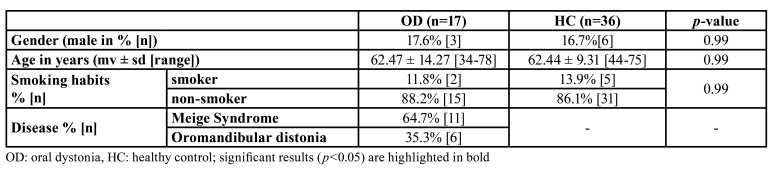
Patient characteristics.

**Table 2:**
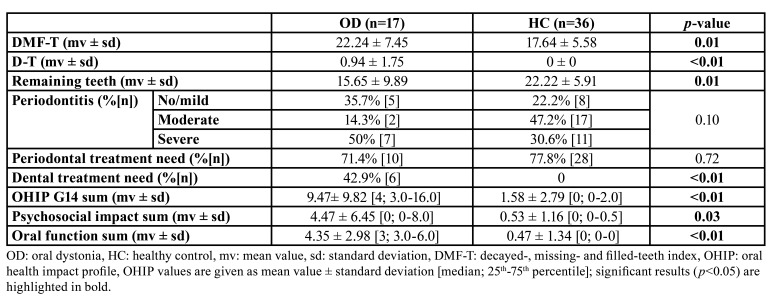
Results of the dental findings and OHIP G14 scores between groups.

- Oral health-related quality of life

Within the OHRQoL assessment, OHIP G14 sum score (9.47± 9.82 vs. 1.58 ± 2.79; *p*<0.01) as well its dimensions psychosocial impact (4.47 ± 6.45 vs. 0.53 ± 1.16; *p*=0.03) and oral function (4.35 ± 2.98 vs. 0.47 ± 1.34; *p*<0.01) were clinically relevant and statistically significant higher in OD compared to HC group (Table 2). Furthermore, the majority of questions (9/14) of the OHIP G14 questionnaire were found to be answered significantly worse in OD group (*p*<0.05, Table 3).

**Table 3:**
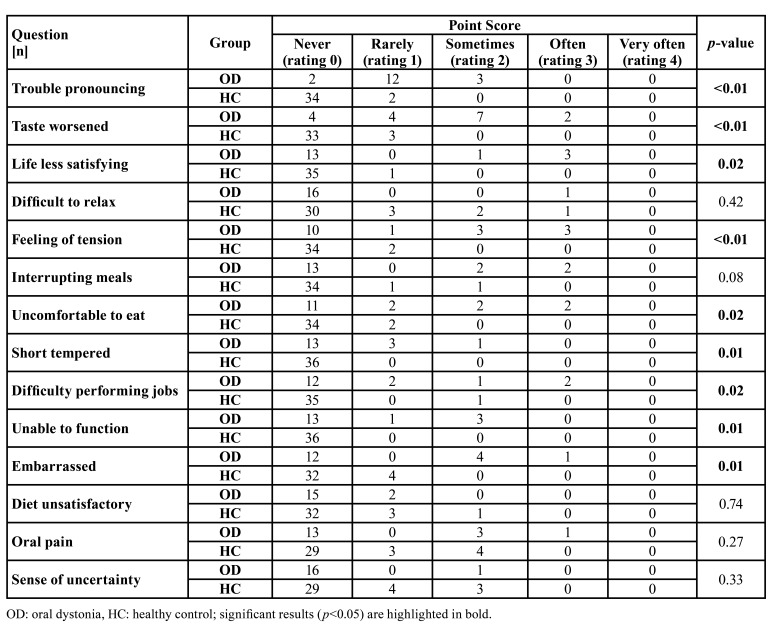
Results of the OHIP G14 questionnaire.

- OHIP G14 and CMD results before and after Botulinum toxin injection

Of the 17 patients with OD, seven patients could be examined again at 3 months after botulinum toxin injection. No significant differences could be detected between baseline and follow-up for OHIP G14 results, mouth opening and Axis II of RDC/TMD (*p*>0.05). However, according to minimal important difference, the OHIP G14 sum score was found to be clinically relevant lower at follow-up appointment (Table 4).

**Table 4:**
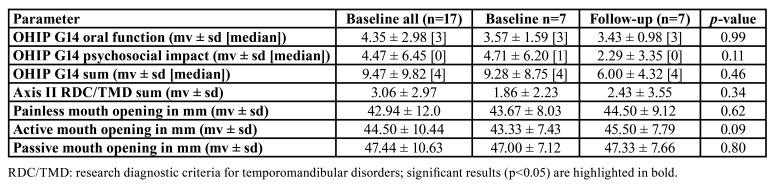
Difference in OHIP G14 and functional findings between baseline and at 3 months after botulinum toxin injection.

## Discussion

- Summary of the main results

Patients with OD in the current study were found to show worse dental health conditions (more caries, less remaining teeth, higher dental treatment need) than HC. Furthermore, the OHRQoL was clinically relevant and statistically significant worse in OD group.

- Comparison with published data

This is the first clinical study that compared oral health and OHRQoL between a group of OD patients with a HC. While for Meige Syndrom no data are available, yet, some studies have examined patients with Oromandibular Dystonia (3-5). These studies reported on a high prevalence of jaw pain (3) or functional limitations (5). Only one study found a higher number of dental problems in patients with Oromandibular Dystonia (4). Because none of the previous examinations focused on oral health findings, especially caries prevalence, remaining teeth and periodontal conditions, no comparable data for this patient group is available. While the higher caries prevalence and related dental treatment need as well as less remaining teeth are reported for the first time in the current study, these results might be of clinical relevance. It is known that the number of missing teeth, especially in posterior area, is related to temporomandibular symptoms (20,21). Thereby, an association between missing teeth and clenching as well as chewing difficulties has been reported (20). Therefore, the higher number of missing teeth could be a potential influential factor on functional complaints in OD patients and should be addressed, accordingly. Regarding caries prevalence and dental treatment need, two factors might be related to the findings of the current study. It is known that caries prevalence is related to oral hygiene measures as well as nutritional behaviour (22,23). Caused by psychosocial and functional complaints due to the dystonia of oral muscles, the patients might be less motivated for personal oral hygiene. Furthermore, limited mouth opening or pain might complicate tooth brushing for the patients. On the other hand, due to problems with chewing and swallowing, a balanced nutrition with fruits and vegeTables might be limited, what could increase the risk of caries. Caries can be an important risk predictor for future tooth loss (24), what could lead to more missing teeth and more functional complains in the future. Accordingly, patients should receive an intensive dental special care, including individual oral hygiene instructions, information and motivation for personal oral hygiene. Moreover, tooth loss should be prevented and a functional rehabilitation under consideration of respective complaints might be considered.

The OHRQoL of OD group was significantly worse than in HC. With regard to the reference values for healthy German population, i.e. 0-4 points depending on dentition, the OD group can also be seen as worse compared to this reference (25). Thereby, total OHIP G14 sum score and both major dimensions oral function and psychosocial impact differed significantly between OD and HC groups. All of these differences were also clinically relevant, following the principle of minimal important difference (16). The OHRQoL is regularly affected by different oral conditions; tooth loss or number of remaining teeth, especially remaining functional occlusal pairs are of relevance in this context, respectively (26,27). Therefore, the reduced number of remaining teeth in OD group could affect their OHRQoL. Moreover, temporomandibular disorders can also lead to a remarkable impairment of OHRQoL (28,29). A large German cohort with temporomandibular disorders was found to have an average OHIP G14 of 14.1, what is higher than in the OD patients in the current study (29). Although oral dystonia is different from temporomandibular symptoms in context of craniomandibular dysfunction, a similar effect could be caused by the functional impairment and jaw pain in context of OD. Moreover, psychosocial impacts related to the underlying OD could be a factor that negatively influences patients OHRQoL. Altogether, the reduced OHRQoL appears to be a further argument for dental special care in patients suffering from OD.

A further sub-aspect was the therapy with botulinum toxin injection, which was examined at three months follow-up in some of the patients. After three months, seven patients were examined again. Thereby, no significant differences between baseline and follow-up were found. However, the OHIP G14 difference was clinically relevant, while missing statistical significance might be caused by the low sample size. No data regarding OHRQoL are available. In general, literature indicates a positive effect of botulinum toxin on complaints of patients with OD (2,5,9-12). In this context, the results of the current study must be seen as preliminary findings, without ability to draw meaningful conclusions on the effect of botulinum toxin injection on OHRQoL of patients with OD.

Altogether, it seems like oral health needs to be supported in patients with OD. It has been stated in literature that dentist needs to be familiar with OD for different reasons; on the one hand, it can develop after dental treatment. Furthermore, it is often misdiagnosed as a dental problem and it may cause important functional complaints and psychosocial disabilities (30). Based on the current study´s findings, dentists’ knowledge on OD is also important because dental health and OHRQoL is impaired in these patients. Moreover, otorhinolaryngologists, head and neck surgeons and maxillo-facial surgeons should know about the dental problems of OD patients and the necessity to foster dental care in these individuals.

- Strengths and limitations

This current study examined oral health and OHRQoL in a group of patients with OD for the first time. The major limitation is the small sample size. However, the included diseases are rare and therefore a large cohort difficult to recruit. With regard to the small sample size, the findings must be seen as preliminary, especially in the follow-up examination. While the examinations were performed by the same experienced and trained dentist, no blinding procedures for baseline as well as follow-up were performed. A potential selection bias of the included participants must be discussed. Because all patients were referred for a specific treatment (special consultation regarding a therapy with botulinum toxin), the representability of the study cohort remains questionable. The examination did not assess explicitly disease related complaints, which could also impact OHRQoL. OD is heterogeneous in the areas they affect; thereby some of the patients with Meige Syndrome might predominantly suffer from blepharospasm with an unclear impact on oral health and OHRQoL, while these patients could have oromandibular manifestations or not. By assessing this issue more in detail, the potential effect of the different forms of OD on OHRQoL might have been more differentiable. A further methodical limitation is the fact that potential underlying diseases, which might affect oral health (e.g. diabetes, rheumatic diseases) were not recorded and considered within analysis. These co-factors might also influence the results and should be recognized in further studies. Furthermore, the form (jaw-opening vs. jaw-closing) as well as localization/manifestation (e.g. tongue involvement) were not considered and analysed, although these parameters could affect oral complaints in OD patients, what might also be relevant for oral health and OHRQoL of the patients. The assessment of botulinum toxin at three months after injection might have been suboptimal, as the effect might have been worn of after this period. Thereby it needs to be considered that the applied OHIP G14 retrospectively assessed OHRQoL or related complaints within the previous month, respectively. Additionally, the effect of botulinum toxin on OHRQoL was just a side issue of the current study, while the focus was set at oral health situation of patients with OD to assess their dental needs. Moreover, general health-related quality of life issues were not considered in the current study. A multicentric, prospective study which should more comprehensively consider disease related issues would be recommendable to confirm the current study´s findings.

## Conclusions

Patients with OD suffer from more dental diseases and show worse OHRQoL than HC. Therefore, dental special care appears recommendable to support patients in their oral hygiene and to prevent caries and tooth loss as good as possible. Everyone, who is involved in the treatment of these patients, including otorhinolaryngologists, head and neck surgeons and maxillo-facial surgeons, should foster their dental care, e.g. by allocation to special dental clinic. The current results and potential benefit of dental special care for patients with OD needs to be validated in prospective studies with a large sample size.
